# Bulbils of Aerial Yam Attenuate Ethanol-Induced Hepatotoxicity in HepG2 Cells through Inhibition of Oxidative Stress by Activation of the Nuclear Factor Erythroid-2-Related Factor 2 Signaling Pathway

**DOI:** 10.3390/nu16040542

**Published:** 2024-02-16

**Authors:** Dong Kwon Yang, Tsendsuren Tungalag, Hyung-Sub Kang

**Affiliations:** 1 Department of Veterinary Pharmacology and Toxicology, College of Veterinary Medicine, Jeonbuk National University, Iksan 54596, Jeollabuk-do, Republic of Korea; dkyang0502@jbnu.ac.kr (D.K.Y.); mgljuuh@jbnu.ac.kr (T.T.); 2 Biosafety Research Institute and Laboratory of Pathology, College of Veterinary Medicine, Jeonbuk National University, Iksan 54596, Jeollabuk-do, Republic of Korea

**Keywords:** bulbil of aerial yam, oxidative stress, reactive oxygen species, ER stress, Nrf2

## Abstract

Bulbil of yam (BY) extract contains various active compounds possessing many pharmacological properties. However, little is known about the effect and underlying mechanism of BY extract on ethanol-induced liver damage. The present study explored the beneficial potential of BY extract on ethanol-induced hepatotoxicity. To evaluate its effectiveness, ethanol-induced HepG2 liver cells were pretreated with BY extract. BY extract effectively rescued cells from ethanol treatment through inhibition of apoptotic cell death as well as inhibiting expression of mitogen-activated protein kinase (MAPK) proteins as stress inducers. BY extract increased the expression of typical antioxidants. Furthermore, BY extract significantly inhibited mitochondrial dysfunction and endoplasmic reticulum (ER) stress, which are major ROS-inducing factors. Finally, as an underlying mechanism of the protective effects of BY extract on ethanol-induced liver damage, it activated Nrf2 protein through translocation from the cytosol to the nucleus, which in turn activated its target oxidative stress suppressor genes. Collectively, our findings demonstrate that BY extract has potential antioxidative effects in ethanol-induced liver cells and contributes to the establishment of a treatment strategy for alcohol-derived liver injuries.

## 1. Introduction

The liver is an important organ for detoxifying various metabolites and synthesizing proteins [1]. For this reason, the liver is vulnerable to various types of injury, such as fibrosis, cirrhosis, and hepatocellular carcinoma, due to exposure to various types of toxins and xenobiotics absorbed from the gastrointestinal tract for the first time [2]. Among them, alcohol causes structural and functional damage to almost every tissue in the body, especially the liver, which is particularly susceptible to damage [2]. Indeed, alcoholic liver disease (ALD), caused by alcohol misuse, is the most common liver disease in the world. According to recent statistics from the World Health Organization (WHO), 3 million people died from alcohol in 2016 worldwide. Among these deaths, digestive diseases accounted for the highest proportion, with 21.3%, and more than 90% of these were associated with ALD, indicating that ALD has become the major cause of death from alcohol [3]. 

Since alcohol is metabolized in the liver, it is susceptible to alcohol [4], causing many forms of ALD, such as fatty liver with cholestasis, chronic liver disease, and alcoholic hepatitis [5]. ALD is a major indication for liver transplantation [6] and results in high mortality [7]. Indeed, alcoholism due to excessive and long-term alcohol consumption seriously damages hepatocytes, which leads to liver fibrosis and cirrhosis and ultimately progresses to hepatocellular carcinoma [8]. Despite specific treatments for severe alcoholic hepatitis using chemical agents or surgical interventions being implemented, such as steroids [9,10], acetylcysteine, antimicrobial and antitumor necrosis factors [11], and liver transplantation [12], the overall prognosis is still very poor. Therefore, it is necessary to identify potential therapeutic agents that can protect the liver from alcohol.

Yam is a monocotyledonous plant classified under the Dioscoreaceae family that forms edible tubercles, such as *D. alata*, *dioscorea*, and *bulbifera* [13]. It is cultivated in some regions, including Africa, Asia, America, the Caribbean, and the Pacific Islands [14]. Importantly, yam possesses many pharmaceutical effects, such as anti-hypercholesteremia [15], antidiabetic [16], antihypertension [17], and antioxidation effects [16,18]. It has also been shown to possess antihyperglycemic effects and has been discovered as a component of foods controlling diabetes [19,20]. Many active compounds in yam have been identified, such as mucin, choline, polyphenols, starches, diosgenin, allantoin, glycoprotein lectin, and dioscorin [21,22,23,24]. Among them, diosgenin has been proven to inhibit the growth of breast cancer cells [14]. Allantoin increases cell proliferation in the body and has been used in cosmetics, painkillers, and skin protection [24]. The glycoprotein isolated from yam has been shown to stimulate the immune system in the body [21]. 

Therapeutic effects of each part of the yam were also elucidated. The leaves of yam have been found to possess several antioxidants [25] and exhibit a cytoprotective effect on HUVEC cells against oxidative stress [26]. The peel of the purple yam has antidiabetic and antioxidant effects on liver cells [16]. In particular, the bulbil of yam has been proven to contain a steroidal saponin phytochemical called diosgenin that possesses antifertility activity [27]. The bulbil of yam, which forms at the leaf axils, has been applied to wounds and has also been used for medicinal purposes to alleviate symptoms of many illnesses, such as dysentery, ulcers, leprosy, and cough [27].

Nuclear factor erythroid-2-related factor 2 (Nrf2) is a transcriptional factor as a critical modulator in diverse cellular functions, such as redox, metabolic homeostasis, DNA repair, and lipid metabolism [28]. It has a crucial role in the inhibition of oxidative stress by activation of target genes possessing antioxidative properties [29]. Therefore, the Nrf2 signaling pathway has beneficial effects against various types of diseases, such as prostate cancer [30], breast cancer [31], and Alzheimer’s diseases [32]. 

The present study demonstrates that the bulbil of aerial yam has a protective effect on HepG2 liver cells against ethanol (EtOH)-induced liver toxicity by inhibiting oxidative stress.

## 2. Materials and Methods

### 2.1. Bulbil of Yam (BY) Extract

Bulbils of yam were purchased from a Korean traditional market (Jeonju, Republic of Korea). A BY ethanol extract was used in this study. Briefly, after washing, BY was dried in a 60 °C incubator and ground into powder using an electric blender. For ethanol extraction, the powdered BY was extracted twice using 80% EtOH (sample: 80% ethanol = 1:8). After filtering, the BY extract was concentrated using a rotary evaporator and freeze-dried. The extraction yield was 18.3%. It was kept at 4 °C for further analyses.

### 2.2. Cell Culture and the Treatment of Aerial Bulbil of Yam and EtOH

The HepG2 cells were obtained from the ATCC (Manassas, VA, USA). Cells were cultured in Dulbecco’s modified Eagle’s medium (GIBCO-BRL, Grand Island, NE, USA) with 10% fetal bovine serum (GIBCO-BRL) and 1% antibiotics (GIBCO-BRL) and maintained in an incubator set at 37 °C and 5% CO_2_. For functional studies, the BY extract with dimethyl sulfoxide (DMSO; Sigma, St. Louis, MO, USA) was pretreated at 10, 20, and 40 µg/mL concentrations for 24 h and then treated with 1 M EtOH (Sigma) for another 24 h to induce hepatotoxicity.

### 2.3. Cell Viability Assay

The BY extract was pretreated with 10, 20, and 40 µg/mL for 24 h to cells (2 × 10^4^), followed by exposure to 1 M EtOH for an additional 24 h, or EtOH alone for 24 h. Cell viability was measured by the 3-[4,5-dimethylthiazol-2-yl]-2,5-diphenyltetrazolium bromide (MTT; Sigma). After adding 0.5 mg/mL MTT solution to each well, they were incubated at 37 °C for 2 h and then treated with DMSO to dissolve the formazan crystals. A plate reader was used to measure the absorbance at 570 nm (Spectra Max M5; Molecular Devices, Sunnyvale, CA, USA).

### 2.4. Hoechst 33342 Staining

Nuclear staining with Hoechst 33342 dye was used to detect apoptotic cells. In brief, the BY extract was pretreated with 10, 20, and 40 µg/mL for 24 h, followed by exposure to 1 M EtOH for an additional 24 h. After fixation with 4% paraformaldehyde for 20 min at room temperature (RT), the cells were stained with 500 ng/mL Hoechst 33342 dye (Thermo Fisher Scientific, Waltham, MA, USA) for 30 min at 37 °C. The nuclei of the cells were observed by fluorescence microscopy (Olympus Corp., Tokyo, Japan).

### 2.5. Measurement of Mitochondrial Membrane Potential (MMP)

MMP was measured using the JC-1 Assay Kit according to the instructions of the manufacturer. In brief, BY extract was pretreated with 10, 20, and 40 µg/mL for 24 h, followed by exposure to 1 M EtOH for an additional 24 h. Cells were then incubated with 10 µg/mL JC-1 dye for 20 min at 37 °C and were further observed by a fluorescence microscope (Oxford Instruments, Oxfordshire, UK). The fluorescence intensity was determined using a fluorescence spectrophotometer (Spectra Max M5) with an excitation wavelength of 550 nm and an emission wavelength of 600 nm as green fluorescence and an excitation wavelength of 485 nm and an emission wavelength of 535 nm as red fluorescence.

### 2.6. Western Blot Analysis

The cells were treated with 1 M EtOH for 24 h after being pretreated with 10, 20, and 40 µg/mL BY extract for 24 h. After cells were lysed with RIPA buffer (LPS Solution, Daejeon, Republic of Korea) containing protease inhibitors (Roche), protein homogenates were separated by sodium dodecyl sulfate–polyacrylamide gel electrophoresis (SDS-PAGE) and transferred to polyvinylidene difluoride (PVDF) membranes. The membranes were incubated with primary antibodies for 5 h at 4 °C and then incubated with secondary antibodies conjugated with horseradish peroxidase for 1 h at RT after blocking in TBST solution with 5% bovine serum albumin for 1 h at RT. The protein bands were visualized with an enhanced chemiluminescence (ECL; Millipore Corp., Billerica, MA, USA) kit and observed with ChemiDoc system (Cleaver Scientific Ltd., Warwickshire, UK). β-actin was used as a loading control. The information concerning the antibodies is shown in the Supplementary Materials. 

### 2.7. Nuclear Fractionation of HepG2 Cells

Cells were treated with 1 M EtOH for 24 h after being pretreated with 10, 20, and 40 µg/mL of the BY extract for 24 h. Fractionation of the nuclei was carried out using the Nuclear Extraction Reagent Kit (Abcam, Cambridge, UK). The protease inhibitors supplemented with lysis buffer A (10 mM HEPES, 1.5 mM MgCl_2_, 10 mM KCl, 0.5 mM DTT, 0.05% NP40, and pH 7.9) were added to cells, followed by incubation for 10 min on ice, and a thorough scraping. The cells were centrifuged at 3000× *g* for 10 min at 4 °C. Then, the supernatants were collected for cytoplasmic fraction. After washing, the nuclear pellet was suspended in lysis buffer B (5 mM HEPES, 1.5 mM MgCl_2_, 0.2 mM EDTA, 0.5 mM DTT, 26% glycerol (*v*/*v*), and pH 7.9) and incubated on ice for 30 min. After centrifugation at 24,000× *g* for 20 min at 4 °C, the supernatant was collected.

### 2.8. Quantitative Real-Time Polymerase Chain Reaction (qRT-PCR)

The BY extract was pretreated with 10, 20, and 40 µg/mL for 24 h, followed by exposure to 1 M EtOH for an additional 24 h. The cDNA was synthesized using the GoScript Reverse Transcription System Kit (Promega Co., Madison, WI, USA), after total RNA was extracted from the cells using the TRIzol reagent (Sigma). qRT-PCR was performed using the kit (Kapa Biosystems, Boston, MA, USA) provided on the machine (Takara Bio. Inc., Shiga, Japan). The expression of each gene was normalized to the expression of the *GAPDH* gene. Primer sequences are shown in the Supplementary Materials.

### 2.9. DPPH Assay for Antioxidant Activity

The 1,1-Diphenyl-2-picrylhydrazyl (DPPH) Antioxidant Assay Kit (ab289847, Abcam, Cambridge, UK) was used to quantify the antioxidant capacity of BY. DPPH working solution (600 µM) with BY extract (10, 20, and 40 µg/mL) was added to each well. The plate was incubated for 10 min at RT, protected from light. Absorbance at 517 nm was determined using a plate reader.

### 2.10. Total Amount of Phenolic Compounds in the BY Extract

The Phenolic Compounds Assay Kit purchased from Abcam was used to measure the total phenolic compounds in the BY extract. The catechol standard solution was used, and vanillic acid was used as a positive control. Furthermore, 100 μL of BY extract solution (10, 20, and 40 μg/mL) and 100 μL of PC assay buffer were added to all the wells to give a final reaction volume of 200 μL. The plate was then incubated for 10 min at RT with gentle shaking. A plate reader was used to determine the absorbance at 480 nm.

### 2.11. Statistical Analysis

The results are presented as the mean ± standard error of the mean (SEM). Statistical analysis was performed using one-way ANOVA and post hoc Bonferroni using Prism 8.02 (GraphPad, San Diego, CA, USA). *p* values less than 0.05 were considered statistically significant.

## 3. Results

### 3.1. BY Extract Rescues HepG2 Liver Cells from EtOH-Induced Cytotoxicity

**Figure 1:**
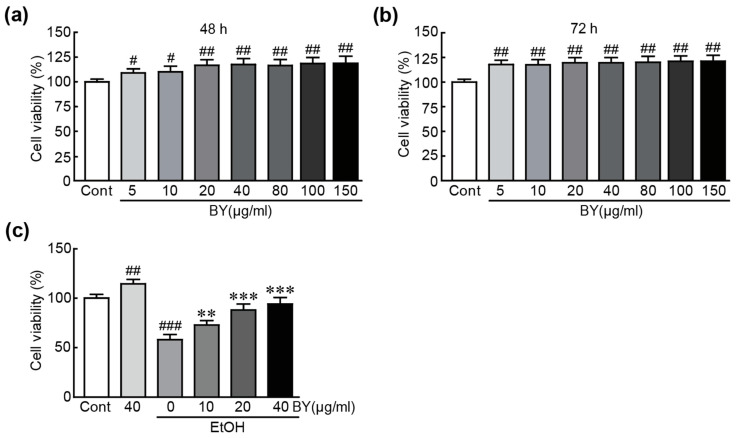
BY extract rescues HepG2 liver cells from EtOH-induced cytotoxicity. Cell viability was checked by MTT assay using the cells treated with BY alone (**a**,**b**) and with BY extract/EtOH-treated (**c**) HepG2 cells. The results are shown as the mean ± SEM in triplicate (*n* = 6). # *p* < 0.05, ## *p* < 0.01, and ### *p* < 0.001 vs. control. ** *p* < 0.01 and *** *p* < 0.001 vs. ethanol-alone-treated group. Cont, control; BY extract, bulbil of yam extract; EtOH, ethanol.

Various concentrations (5~150 µg/mL) of BY extract were treated for 48 h and 72 h to test the cytotoxicity of BY extract on HepG2 cells. The results showed that no cytotoxicity was shown in any concentration of BY extract for both 48 h and 72 h. Therefore, we chose three kinds of concentrations (10, 20, and 40 of µg/mL) of BY extract for further studies (Figure 1a,b). To determine the protective effect of BY extract against EtOH-induced cytotoxicity, cells were pretreated with 10, 20, and 40 µg/mL of BY extract, followed by 1 M EtOH for another 24 h. An MTT assay was used to check their viability. When treated with 1 M EtOH alone, the viability of cells was significantly decreased, with a value of 58.0% compared with that of control cells (Figure 1). Otherwise, more of the cells pretreated with BY extract survived than the EtOH-alone-treated cells in a dose-dependent manner (73%, 88%, and 94% survival rates in 10, 20, and 40 µg/mL BY extract-pretreated cells, respectively, compared with those in control cells; Figure 1). These results indicate that BY extract has a preventative effect on EtOH-induced cytotoxicity in HepG2 cells.

### 3.2. BY Extract Suppresses EtOH-Induced Apoptotic Responses in HepG2 Cells

**Figure 2:**
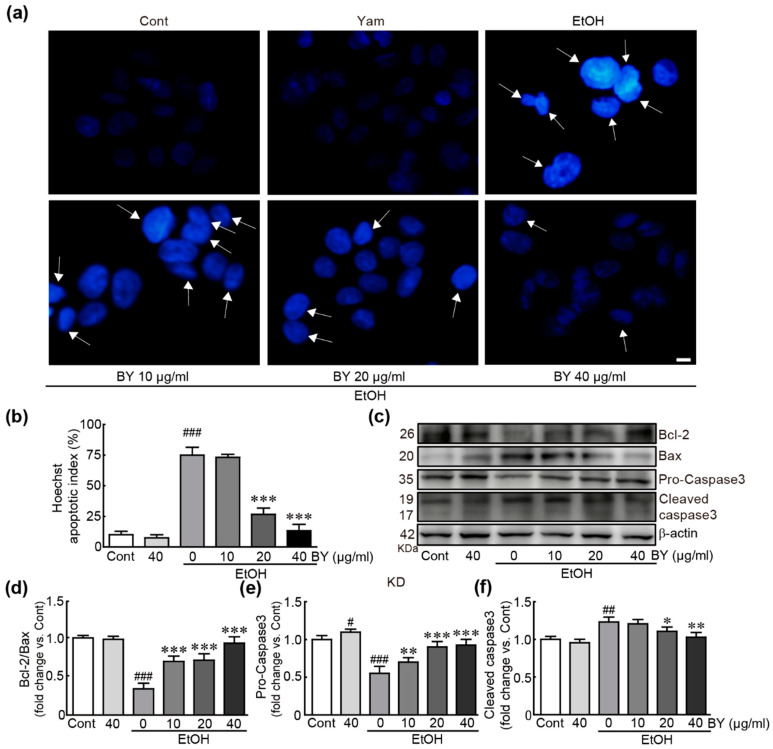
BY extract suppresses EtOH-induced apoptotic cell death in HepG2 cells. (**a**) Hoechst staining was performed for nuclear staining using cells treated with BY extract and/or EtOH. The arrow indicates apoptotic cells with nuclear condensation. (**b**) The apoptotic index was shown as the percentage of apoptotic cells vs. total cells. A total of 50 cells were counted in at least 10 fields. Western blot analysis for apoptosis-related proteins (**c**) and the densities of the ratio of Bcl-2/Bax (**d**) and pro- (**e**) and cleaved (**f**) caspase-3 proteins. The results are shown as the mean ± SEM in triplicate (*n* = 6). # *p* < 0.05, ## *p* < 0.01, ### *p* < 0.001 vs. control. * *p* < 0.05, ** *p* < 0.01, and *** *p* < 0.001 vs. ethanol-alone-treated group. Cont, control; BY extract, bulbil of yam extract; EtOH, ethanol.

To determine whether BY extract can suppress apoptotic responses due to EtOH-induced hepatotoxicity in HepG2 cells, we first checked nuclear condensation using Hoechst staining (Figure 2a). The results showed that the cells undergoing apoptosis were dramatically increased in the EtOH-only-treated cells (75% apoptotic cell proportion; Figure 2b). Otherwise, the pretreatment with BY extract dramatically suppressed the apoptosis in 20 and 40 µg/mL EtOH-treated cells (25%, and 13% apoptotic cell proportion in 20 and 40 µg/mL BY extract-pretreated cells, respectively; Figure 2b). Next, we determined the expression of several apoptosis-related proteins, such as Bcl-2, Bax, and caspase 3, using Western blot analysis. The results showed that the amount of Bcl-2 protein possessing the ability to inhibit the apoptotic signaling pathway was significantly decreased; otherwise, the Bax protein that causes apoptosis was significantly increased in EtOH-only-treated cells (0.3-fold change in Bcl-2/Bax, compared with that in control cells; Figure 2c,d). Pro-caspase 3 as an inactive form of this protein was decreased and cleaved-caspase 3 as an active form of this protein was increased in the EtOH-alone-treated cells, indicating that caspase 3 as a pro-apoptotic protein was activated due to the EtOH treatment (0.5- and 1.2-fold changes in pro- and cleaved-caspases 3 in EtOH-alone-treated cells, respectively, compared to the control cells; Figure 2c,d). Additionally, in the BY extract-alone-treated cells, pro-caspase 3 was significantly increased and cleaved-caspase 3 tended to be decreased (Figure 2c,e,f). Notably, these changes were dramatically ameliorated by pretreatment with BY extract in a dose-dependent manner. Therefore, these results indicate that BY extract effectively attenuates EtOH-induced apoptotic responses in HepG2 cells.

### 3.3. BY Extract Inhibits the Activation of MAPK Signaling Induced by EtOH in HepG2 Cells

**Figure 3:**
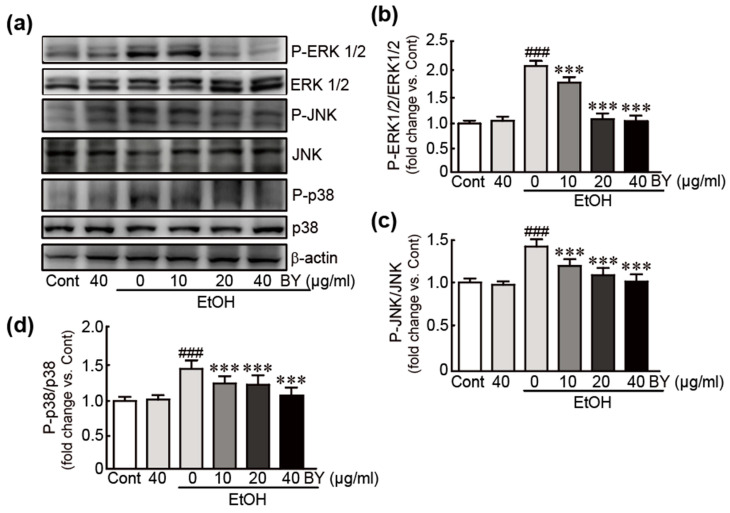
BY extract inhibits the activation of MAPK signaling induced by EtOH in HepG2 cells. Western blot analysis for total and phospho-MAPK proteins using cells treated with BY extract and/or EtOH (**a**) and the densities of the ratios of p-ERK/ERK (**b**), p-JNK/JNK (**c**), and p-p38/p38 (**d**). The results are shown as the mean ± SEM in triplicate (*n* = 6). ### *p* < 0.001 vs. control. *** *p* < 0.001 vs. ethanol-alone-treated group. Cont, control; BY extract, bulbil of yam extract; EtOH, ethanol.

The MAPK signaling pathway has been demonstrated to be closely associated with EtOH-induced liver toxicity [33]. Therefore, we investigated whether BY extract attenuates the activation of the MAPK signaling pathway in EtOH-treated HepG2 cells. Thus, Western blot analysis was performed against typical MAPK proteins, including ERK1/2, JNK, and p38. The results showed that phosphorylation of ERK1/2, JNK, and p38 proteins was significantly increased in EtOH-alone-treated cells (2.1-, 1.5-, and 2.1-fold increases in p-ERK1/2, p-JNK, and p-p38, respectively, compared to control cells; Figure 3), indicating that they were activated due to EtOH treatment. Otherwise, BY extract pretreatment in EtOH-treated cells significantly inhibited the phosphorylation of these proteins. Therefore, these results indicate that BY extract effectively suppresses the MAPK signaling pathway in hepatotoxicity by EtOH.

### 3.4. BY Extract Inhibits EtOH-Induced Oxidative Stress in HepG2 Cells

**Figure 4:**
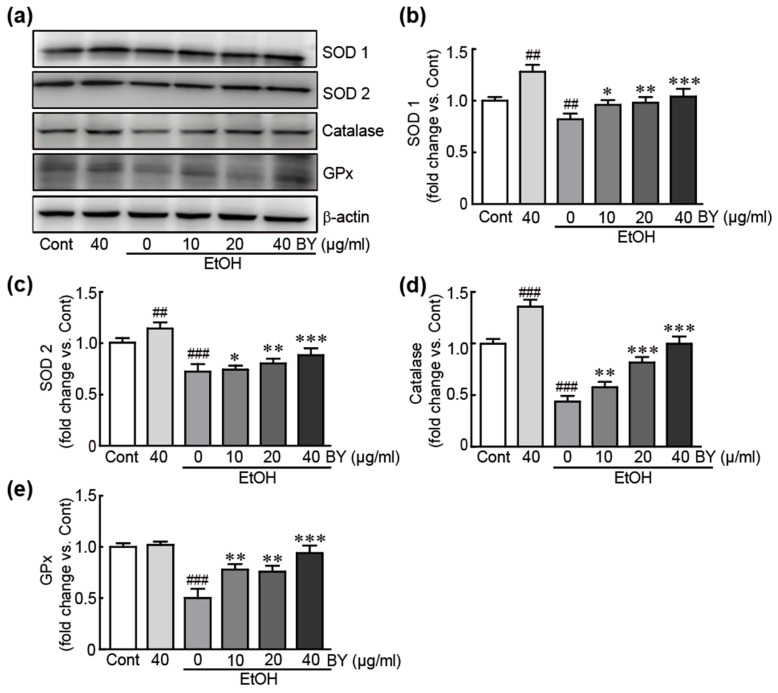
BY extract inhibits EtOH-induced oxidative stress in HepG2 cells. Western blot analysis for antioxidants, including SOD1, SOD2, catalase, GPx using cells treated with BY extract and/or EtOH (**a**) and the densities of SOD1 (**b**), SOD2 (**c**), catalase (**d**), and GPx (**e**) proteins were calculated with HD9 imaging software. The results are shown as the mean ± SEM in triplicate (*n* = 6). ## *p* < 0.01 and ### *p* < 0.001 vs. control. * *p* < 0.05, ** *p* < 0.01, and *** *p* < 0.001 vs. ethanol-alone-treated group. Cont, control; BY extract, bulbil of yam extract; EtOH, ethanol; SOD 1, superoxide dismutase 1; SOD 2, superoxide dismutase 2; GPx, glutathione peroxidase.

To determine whether BY extract could inhibit the oxidative stress due to EtOH-induced hepatotoxicity, the levels of typical antioxidants, such as SOD1,2, catalase, and GPx, were checked using Western blot analysis. These antioxidants were dramatically decreased in EtOH-alone-treated cells; otherwise, when pretreated with BY extract, the levels were significantly increased compared to EtOH-alone-treated cells in a dose-dependent manner. In particular, pretreatment with 40 µg/mL BY extract dramatically increased them with similar levels to those in control cells (Figure 4). Additionally, SOD1, 2, and catalase were significantly increased in BY extract-alone-treated cells (Figure 3a–d). Therefore, BY extract rescues HepG2 cells from EtOH-induced hepatotoxicity.

### 3.5. BY Extract Attenuates Mitochondrial Dysfunction Induced by EtOH in HepG2 Cells

**Figure 5:**
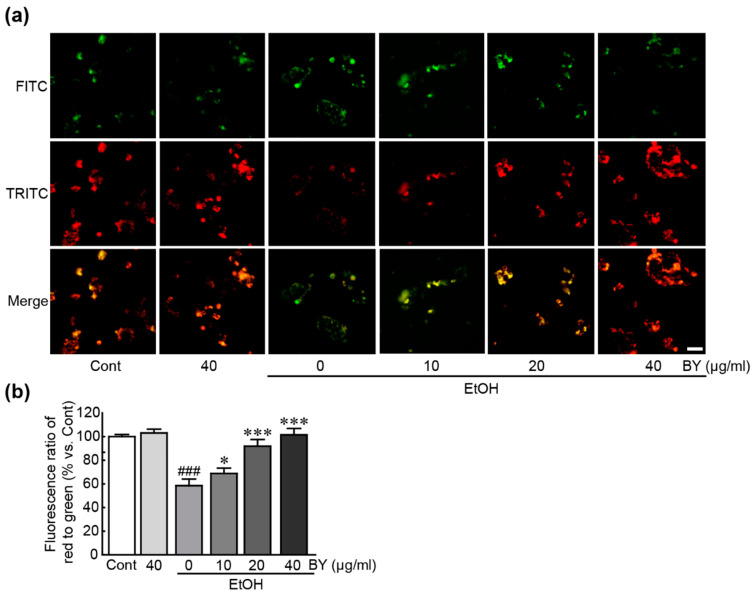
BY extract attenuates EtOH-induced mitochondrial dysfunction in HepG2 cells. (**a**) MMP was determined by JC-1 staining assay using cells treated with BY extract and/or EtOH. Red and green fluorescences represent normal and disrupted MMPs, respectively. (**b**) The percentage ratio of red/green fluorescence intensities vs. control group was measured. The results are shown as the mean ± SEM in triplicate (*n* = 6). ### *p* < 0.001 vs. control. * *p* < 0.05 and *** *p* < 0.001 vs. ethanol-alone-treated group. Cont, control; BY extract, bulbil of yam extract; EtOH, ethanol; MMP, mitochondrial membrane potential.

To evaluate the effect of BY extract on the pathological changes in mitochondria due to EtOH-induced hepatotoxicity, mitochondrial function was evaluated through JC-1 dye immunostaining with an MMP detector using EtOH-alone-treated cells and BY extract (10, 20, and 40 µg/mL)-pretreated cells. The results showed that green fluorescence was predominant in the EtOH-alone-treated cells, but red fluorescence prevailed in the control cells (58.5% decrease in the ratio of red/green fluorescence compared to control cells; Figure 5). Therefore, MMP was disrupted by EtOH. In particular, red fluorescence is prominent in BY extract-pretreated cells (Figure 5), indicating that BY extract protected MMP against EtOH-induced hepatotoxicity. Notably, treatment with a high concentration of BY extract (40 µg/mL) has shown a similar level to that in control cells. Therefore, BY extract has been proven to attenuate mitochondrial dysfunction caused by EtOH-induced hepatotoxicity in HepG2 cells.

### 3.6. BY Extract Inhibits the Activation of Endoplasmic Reticulum (ER) Stress Induced by EtOH in HepG2 Cells

**Figure 6:**
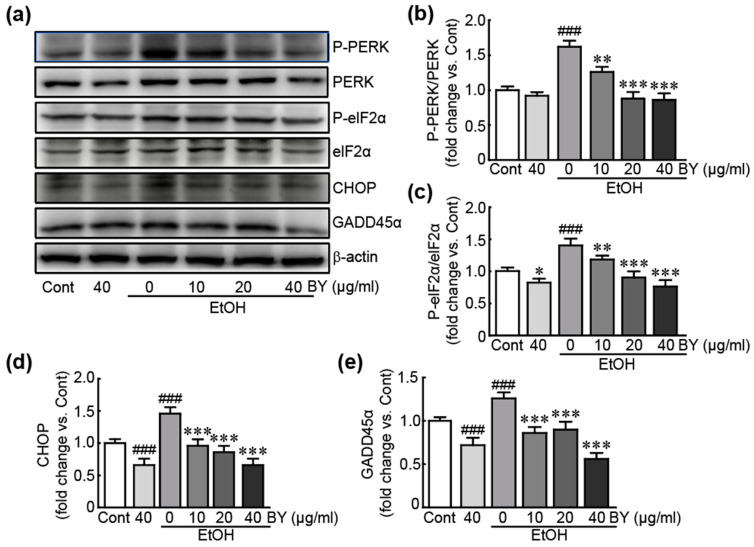
BY extract inhibits the activation of EtOH-induced ER-related stress in HepG2 cells. Western blot analysis for ER-stress-related proteins using cells treated with BY extract and/or EtOH (**a**) and the densities of the ratios of p-PERK/PERK (**b**), p-eIF2α/eIF2α (**c**), ATF (**d**), and GADD45α (**e**) in triplicate (*n* = 6). ### *p* < 0.001 vs. control. * *p* < 0.05, ** *p* < 0.01, and *** *p* < 0.001 vs. ethanol-alone-treated group. Cont, control; BY extract, bulbil of yam extract; EtOH, ethanol.

We further evaluated the effects of BY extract on ER stress responses due to ER-related stress, considered as an incidence of oxidative stress and mitochondrial dysfunction. Western blot analysis was performed against typical ER stress signaling-related proteins, including PERK, eIF2α, CHOP, and GADD45α. First, phosphorylated PERK and eIF2α were dramatically increased in EtOH-alone-treated cells (1.6- and 1.4-fold increases in p-PERK and p-eIF2α in EtOH-alone-treated cells, respectively, compared to control cells; Figure 6a–c). Additionally, the expression levels of CHOP and GADD45α proteins were significantly increased in EtOH-alone-treated cells (1.5- and 1.3-fold increases in CHOP and GADD45α in EtOH-only-treated cells, respectively, compared to control cells; Figure 6a,d,e). In the BY extract-alone-treated cells, the expression levels of phosphorylated eIF2α, CHOP, and GADD45α proteins were significantly decreased (Figure 6). These results indicate that EtOH activates ER stress. Conversely, pretreatment with BY extract significantly inhibited the activation or downregulated these proteins in EtOH-treated cells. Therefore, BY extract effectively attenuates the activation of ER-related stress by EtOH in HepG2 cells.

### 3.7. BY Extract Activates Nrf2-Related Signaling Pathway in EtOH-Treated HepG2 Cells

**Figure 7:**
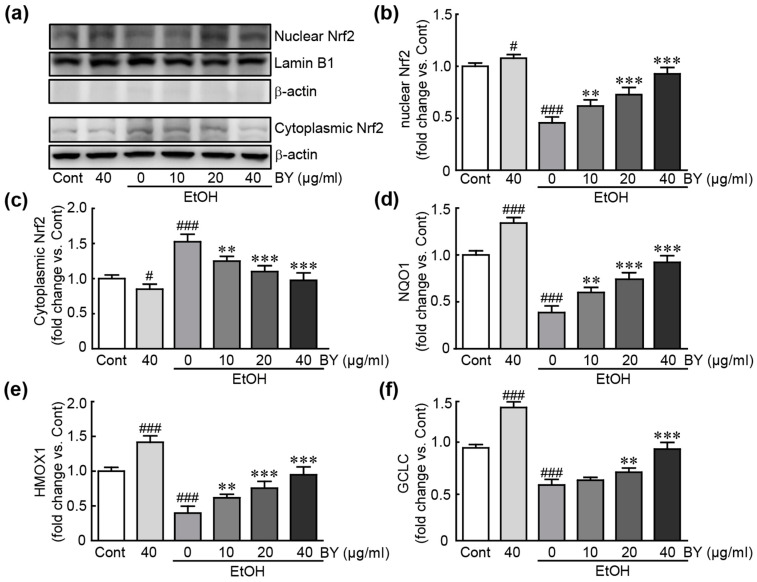
BY extract activates the Nrf2-related signaling pathway in EtOH-treated HepG2 cells. Western blot analysis for Nrf2 after nuclear fractionation using cells treated with BY extract and/or EtOH (**a**) and the density of nuclear (**b**) and cytoplasmic Nrf2 (**c**) (n = 6). qRT-PCR analysis of Nrf2 target genes, including *NQO1* (**d**), *HMOX1* (**e**), and *GCLC* (**f**) in triplicate (*n* = 6). # *p* < 0.05 and ### *p* < 0.001 vs. control. ** *p* < 0.01 and *** *p* < 0.001 vs. ethanol-alone-treated group. Cont, control; BY extract, bulbil of yam extract; EtOH, ethanol; NQO1, NAD(P)H quinone oxidoreductase; HMOX1, heme oxygenase 1; GCLC, glutamate-cysteine ligase catalytic subunit.

To elucidate the underlying mechanism of the antioxidative effects of BY extract in EtOH-induced hepatotoxicity, we determined whether the Nrf2 signaling pathway with potential antioxidative properties [34] is activated in EtOH-treated HepG2 cells. Western blot analysis demonstrated that the Nrf2 protein increased when pretreated with BY extract, while it decreased in EtOH-alone-treated cells, indicating that the Nrf2 protein is activated with BY extract pretreatment (Figure 7a,b). In addition, in qRT-PCR analysis of target downstream genes (*NQO1*, *HMOX1*, and *GCLC*) with inhibitory effects against oxidative stress, their expression was dramatically decreased in EtOH-alone-treated cells; otherwise, pretreatment with BY extract significantly restored the expression of these genes (Figure 7c–e). Therefore, BY extract inhibits oxidative stress through the Nrf2-related signaling pathway in EtOH-induced hepatotoxicity.

### 3.8. DPPH Radical Scavenging Activity and Total Phenolic Compounds in BY Extract

**Figure 8:**
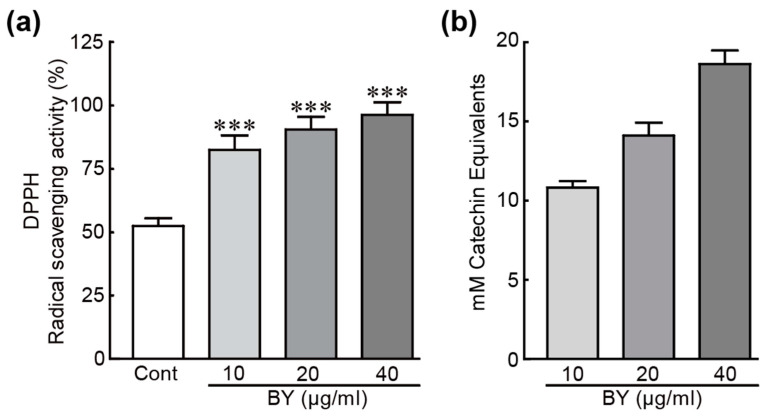
DPPH radical scavenging activity and total phenolic compounds in BY extract. DPPH-radical scavenging activity (**a**) and total phenolic compounds (**b**) were measured at various concentrations of BY extract (10, 20, and 40 µg/mL) (*n* = 6). *** *p* < 0.001 vs. control. Cont, control; BY extract, bulbil of yam extract.

DPPH, as an organic free radical, has been widely used for determining the antioxidative properties of plant extracts or compounds [35]. Therefore, a DPPH assay was performed to elucidate the ability of DPPH radical scavenging to determine the antioxidant activity of BY extract. As shown in Figure 8, the DPPH radical scavenging activities of BY extract dramatically increased in a dose-dependent manner compared to that of control (82.4%, 90.4%, and 96.2% increase in 10, 20, 40 µg/mL BY extract, respectively; Figure 8a). Phenolic compounds play important roles in redox ability, which is essential for antioxidant activity [36]. Therefore, we determined the total phenol contents in BY extract using a commercially available kit. Total phenol contents were determined from the standard curve with different concentrations of catechin as a standard compound and were expressed as mM catechin equivalents (CE). Total phenol contents contained 10, 20, and 40 µg/mL BY extract were 10.8, 14.1, and 18.6 mM CE, respectively (Figure 8b).

## 4. Discussion

Much evidence has been accumulated that yam possesses therapeutic potential against various diseases, such as inflammation, gastrointestinal disease, diabetes, and cancer. A previous study reported that the bulbils of yam possess potent analgesic and anti-inflammatory activities [37]. However, the effects of bulbil of yam on other diseases need to be studied. Therefore, in the present study, we pursued the potential preventative properties of bulbil of yam on EtOH-induced hepatotoxicity using HepG2 cells.

First, we observed that the BY extract dramatically increased the viability of EtOH-treated cells. We then determined whether BY extract could inhibit apoptotic cell death as a major influencing factor in cell viability. BY extract has been proven to inhibit DNA damage from nuclear staining and also to attenuate the increased levels of apoptotic signaling-related proteins by EtOH. Therefore, BY extract effectively rescues HepG2 liver cells from EtOH. Additionally, the activities of typical MAPK proteins, including ERK, JNK, and p38, were also measured in BY extract-pretreated cells. MAPK signaling is involved in various cellular functions, such as proliferation, differentiation, and apoptosis [38]. Furthermore, it plays an important role in contributing to the pathogenesis of alcoholic liver injury by promoting inflammation, oxidative stress, etc. [39]. Thus, BY extract effectively suppresses the activation of these MAPK proteins. 

Oxidative stress is closely associated with the development of alcoholic liver diseases [40]. Alcohol metabolism primarily occurs in the liver through oxidative enzymatic pathways. The first classical pathway is mediated by alcohol dehydrogenase, which converts alcohol into acetaldehyde [41]. The acetaldehyde is further metabolized to acetate. The second major pathway for alcohol degradation is catalyzed by the 2E1 isoform of the cytochrome P450 (CYP2E1) [42]. Notably, this metabolic pathway leads to ROS generation, resulting in oxidative stress, which further provokes liver damage. Previous studies using CYP2E1-overexpressed HepG2 cells or knock-in mice demonstrated that oxidative stress is increased, lipid peroxidation and apoptosis occur in cells, and elevated hepatic steatosis and liver injury occur in hepatic tissues after the administration of alcohol [43,44]. On the other hand, CYP2E1 knockout mice showed decreased oxidative stress and were protective against alcohol-induced liver injury [45]. In this regard, we elucidated the preventative effects of BY extract against oxidative stress in EtOH-treated cells. Western blot analysis demonstrated that pretreatment with BY extract protects typical antioxidants, including SOD1, 2, catalase, and GPx, in EtOH-exposed HepG2 cells, indicating that BY extract could block oxidative stress by increasing levels of antioxidants. Additionally, we also sought to verify the ability of BY extract to suppress mitochondrial dysfunction. MMP, as an important indicator of mitochondrial damage, was disrupted due to EtOH treatment; in other respects, BY extract was proven to preserve MMP from EtOH-induced hepatotoxicity. Mitochondria are important organelles for energy metabolism, calcium homeostasis, cell survival, and apoptosis [46]. Among them, the principal function of mitochondria is ATP synthesis through oxidative phosphorylation (OXOPHOS) as an electron transfer chain reaction (ETC) [47]. ETC has been considered the predominant route for ROS generation [48]. Therefore, mitochondria are an important source of ROS generation within the cell. Although the physiological level of ROS generation is necessary and involved in the regulation of many cellular activities, ROS accumulates and causes multiple cellular disruptions involved in the development of multiple diseases when its production exceeds the antioxidant capacity due to mitochondrial dysfunction [46]. Accordingly, our results demonstrated that EtOH treatment caused mitochondrial dysfunction, contributing to oxidative stress. However, pretreatment with BY extract effectively attenuated the mitochondrial dysfunction.

Since hepatocytes produce many secreted proteins, including albumin and lipoproteins, to perform important metabolic, secretory, or excretory functions, a lot of protein synthesis and folding are required [49]. Therefore, given that the function of ER is protein synthesis and processing, hepatocytes are enriched in ER and susceptible to ER perturbation and stress [50]. Upon ER stress due to the excessive accumulation of unfolded and misfolded proteins, the unfolded protein response (UPR) is activated as an adaptive response to maintain ER homeostasis [51]. However, when ER stress persists, the UPR triggers cell death [52]. Furthermore, increasing evidence indicates that ER stress has a crucial role in the pathogenesis of liver diseases, such as nonalcoholic fatty liver disease, cholestatic liver disease, hepatocellular carcinoma, and alcoholic liver disease [49]. Herein, ER stress-related proteins were activated or increased in EtOH-treated cells; in other respects, the BY extract significantly attenuated the activation of or increased these proteins, suggesting that inhibition of ER stress mediates the preventative effect of BY extract against EtOH-induced hepatotoxicity.

To elucidate the underlying mechanism of the preventative effects of BY extract on EtOH-induced hepatotoxicity, we sought to determine the involvement of the Nrf2 signaling pathway. Nrf2, as a transcriptional factor, has a variety of potential properties in cell defense and homeostasis against oxidative stress, detoxification, and inflammation [53]. Nrf2 is normally present in the cytoplasm, but when activated, it moves to the nucleus and acts as a transcriptive activator for various genes with antioxidative functions, such as *NQO1*, *SOD*, *HMOX1*, *GCLC*, and *GCLM* [54]. Therefore, it specifically functions as a defense against oxidative stress. Furthermore, the protective roles of Nrf2 activation in the pathogenesis of liver diseases, especially alcoholic liver disease, have been extensively investigated [55]. A previous study demonstrated that the upregulation of Nrf2 inhibited ROS production and apoptosis in EtOH-exposed mice [56]. Moreover, another study revealed that Nrf2-mediated cytoprotective enzymes could ameliorate alcohol-induced liver steatosis both in in vivo and in vitro models [57]. Therefore, NRF2 shows potential therapeutic use for ALD treatment. Consistent with these studies, our results showed that BY extract translocated the Nrf2 protein from the cytosol to the nucleus and then activated various target genes possessing antioxidative properties. 

Finally, we demonstrated that BY extract possesses high antioxidant activity by measuring DPPH scavenging activity. Previous studies have demonstrated that many polyphenols contained BY, such as gallic acid, epicatechin, catechins, quercetin, and kaempferol, have been identified, which possess antioxidant activities [58,59]. In this study, we demonstrated that BY extract contains a high total phenolic content, which contributes to its antioxidant activity in EtOH-treated cells. This study first describes the preventative effects of BY extract on EtOH-induced hepatic cells. 

## 5. Conclusions

In conclusion, this study suggests that BY extract, possessing potential antioxidant properties, attenuates alcohol-induced hepatotoxicity via phenolic compounds possessing antioxidative properties and the activation of the Nrf2 signaling pathway. Therefore, we propose that BY extract can be used as a potential therapeutic for the prevention or treatment of alcoholic liver diseases.

## Data Availability

Data are contained within the article.

## Supplementary Materials

The following supporting information can be downloaded at: https://www.mdpi.com/article/10.3390/nu16040542/s1.
