# Supplementary material for: Bulbils of Aerial Yam Attenuate Ethanol-Induced Hepatotoxicity in HepG2 Cells through Inhibition of Oxidative Stress by Activation of the Nuclear Factor Erythroid-2-Related Factor 2 Signaling Pathway
Source: Nutrients. 2024 Feb 16;16(4):542. doi: 10.3390/nu16040542 (PMC10892442; doi:10.3390/nu16040542)
Supplement: Supplementary file 1 [file nutrients-16-00542-s001.zip › nutrients-2819733-supplementary.pdf]

Supplementary Table S1. List of antibodies

Antibody	Catalog number	Working dilution	Company
Bcl-2	sc-492	1:1,000	Santa Cruz Biotechnology
Bax	#2772	1:1,000	Cell signaling
pro-caspase 3	#9665	1:1,000	Cell signaling
cleaved caspase 3	#9664	1:1,000	Cell signaling
SOD1	sc-101523	1:1,000	Santa Cruz Biotechnology
SOD2	#1341	1:1,000	Cell signaling
catalase	#14097	1:1,000	Cell signaling
PERK	#3192	1:1,000	Cell signaling
p-PERK	Ab192591	1:1,000	Abcam
eIF2α	#9722	1:1,000	Cell signaling
p-eIF2α	#3597	1:1,000	Cell signaling
CHOP	#2895	1:1,000	Cell signaling
GADD45α	#4632	1:1,000	Cell signaling
ERK1/2	#9102	1:1,000	Cell signaling
p-ERK1/2	#9101	1:1,000	Cell signaling
JNK	#9252	1:1,000	Cell signaling
p-JNK	#3251	1:1,000	Cell signaling
p38	#9212	1:1,000	Cell signaling
p-p38	#9211	1:1,000	Cell signaling
Nrf2	#12721	1:1,000	Cell signaling
β-actin	sc-47778	1:1,000	Santa Cruz Biotechnology

Supplementary Table S2. Specific primer sequences for qRT-PCR

Genes	Accession No.	Primers	
NQO1	NM_017000	forward	5′-TGAGCCCGGATATTGTAGCTGA-3′
		reverse	5′-GCATACGTGTAGGCGAATCCTG-3′
HMOX1	NM_012580	forward	5′-ATTTGTCCGAGGCCTTGAA-3′
		reverse	5′-CCAGGGCCGTATAGATATGGTA-3′
GCLC	NM_012815.2	forward	5′-GTGGACACCCGATGCAGTA-3′
		reverse	5′-CTTGTAGTCAGGATGGTTTGCAATA-3′
GAPDH	NM_017008	forward	5′-CTCTACCCACGGCAAGTTC-3′
		reverse	5′-GCCAGTAGACTCCACGACATA-3′
18S rRNA (Internal control)	NR_003278.3	forward	5′-GCAATTATTCCCCATGAACG-3′
		reverse	5′-GGCCTCACTAAACCATCCAA-3′

TNFα; tumor necrosis factor α, IL-1β; interleukin-1β, IL-6; interleukin-6, 18S; 18S ribosomal RNA
